# Concurrent Exposure of Neutralizing and Non-neutralizing Epitopes on a Single HIV-1 Envelope Structure

**DOI:** 10.3389/fimmu.2019.01512

**Published:** 2019-07-05

**Authors:** Krishanu Ray, Meron Mengistu, Chiara Orlandi, Marzena Pazgier, George K. Lewis, Anthony L. DeVico

**Affiliations:** ^1^Institute of Human Virology, University of Maryland School of Medicine, Baltimore, MD, United States; ^2^Department of Biochemistry and Molecular Biology, University of Maryland School of Medicine, Baltimore, MD, United States; ^3^Department of Medicine, University of Maryland School of Medicine, Baltimore, MD, United States; ^4^Department of Microbiology and Immunology, University of Maryland School of Medicine, Baltimore, MD, United States

**Keywords:** single HIV-1 virion, epitope exposure, neutralizing and non-neutralizing epitopes, two-color coincidence fluorescence correlation spectroscopy (FCS), FRET-FCS

## Abstract

The trimeric envelope spikes on the HIV-1 virus surface initiate infection and comprise key targets for antiviral humoral responses. Circulating virions variably present intact envelope spikes, which react with neutralizing antibodies; and altered envelope structures, which bind non-neutralizing antibodies. Once bound, either type of antibody can enable humoral effector mechanisms with the potential to control HIV-1 infection *in vivo*. However, it is not clear how the presentation of neutralizing vs. non-neutralizing epitopes defines distinct virus populations and/or envelope structures on single particles. Here we used single-virion fluorescence correlation spectroscopy (FCS), fluorescence resonance energy transfer (FRET), and two-color coincidence FCS approaches to examine whether neutralizing and non-neutralizing antibodies are presented by the same envelope structure. Given the spatial requirements for donor-acceptor energy transfer (≤10 nm), FRET signals generated by paired neutralizing and non-neutralizing fluorescent Fabs should occur via proximal binding to the same target antigen. Fluorescent-labeled Fabs of the neutralizing anti-gp120 antibodies 2G12 and b12 were combined with Fabs of the non-neutralizing anti-gp41 antibody F240, previously thought to mainly bind gp41 “stumps.” We find that both 2G12-F240 and/or b12-F240 Fab combinations generate FRET signals on multiple types of virions in solution. FRET efficiencies position the neutralizing and non-neutralizing epitopes between 7.1 and 7.8 nm apart; potentially fitting within the spatial dimensions of a single trimer-derived structure. Further, the frequency of FRET detection suggests that at least one of such structures occurs on the majority of particles in a virus population. Thus, there is frequent, overlapping presentation of non-neutralizing and neutralizing epitope on freely circulating HIV-1 surfaces. Such information provides a broader perspective of how anti-HIV humoral immunity interfaces with circulating virions.

## Introduction

Intensive efforts are underway to develop preventive vaccines and therapeutic strategies based on humoral immunity against the HIV-1 envelope (Env). Such efforts logically consider directing antibody responses toward replication competent viral particles. Success in this regard demands an understanding of the epitope patterns expressed by virions and virus populations. On HIV-1 particles, the virus envelope spike is a heavily glycosylated trimer of three heterodimers containing gp120 surface subunits and gp41 transmembrane proteins. Gp120 binds the host cell receptor CD4 and a co-receptor, which triggers gp41 to mediate membrane fusion and viral entry. These antigens exhibit high variability in sequence and structure, driven by and allowing escape from immune pressure (1–13). At the same time, Env antigens can express highly conserved epitopes of various types, e.g., within glycan domains; within the CD4 or co-receptor binding sites; or on gp41. These epitopes are highly attractive targets for vaccine design as human antibodies (bNAbs) against them can be very broadly neutralizing (14–16) and provide potent sterilizing protection against SHIV challenge in macaque infection models (17–19).

The most highly conserved, functional and immutable Env epitopes are not neutralizing, often because they are structurally occluded on free trimers (20–22). Many of these epitopes are exposed as a consequence of natural virus-cell attachment mechanisms (21–23) but it is unclear how they are presented by free virions. One possibility is that non-neutralizing epitopes are expressed on “aberrant,” non-functional envelope structures (24–26). Even if disconnected from productive attachment and entry processes, such structures could still mediate antiviral immunity if they appear on replication competent virions. Previous studies showed that non-neutralizing humoral responses directed against non-HIV antigens placed on functional virions mediated protection from SIV or HIV-1 infection in macaque and humanized mouse models, respectively (27–31). Thus, the presence and nature of any Env structure appearing in a virus population warrants careful evaluation in the context of antiviral immunity.

Numerous attempts have been made to characterize the prevalence of non-neutralizing vs. neutralizing epitope presentation in populations of HIV-1 virions and/or to partition HIV-1 virion populations into replication-competent vs. non-functional particles based on differential epitope presentations (9, 25, 26, 32–35). Such work has relied heavily on some manner of virion capture by anti-Env antibodies bound to a substrate. In general, findings from this approach have suggested that virus preparations can contain subpopulations of virions presenting variable mixtures of neutralizing and non-neutralizing epitopes. Subpopulations favoring non-neutralizing epitopes (presumably harboring a large amount of defective or degraded envelope) tend to be poorly- or non-infectious. Although useful, capture systems inform the nature of virions after some sort of adsorption procedure. Associated caveats include altered immunoreactivity patterns caused by the process of substrate attachment; altered virion characteristics caused by the capture manipulations. With some techniques, captured material may represent aggregates of particles as well as single virions. Moreover, capture methods cannot directly reveal whether individual virions present neutralizing and non-neutralizing epitopes on common surface structures, and/or show how frequently such scenarios occur within a virion population.

Previously we developed an analytical method based on fluorescence correlation spectroscopy (FCS) that allows the direct evaluation of mAb binding to HIV-1 virions continuously in solution (36). More recently we adapted this method to enable dual color detection of two different fluorescent-labeled mAbs bound to a single virion as well as detection of Förster resonance energy transfer (FRET) from fluorophores closely localized on single Env spikes. These tools can be used to investigate the relative presentation of neutralizing and non-neutralizing epitopes in virion populations and/or single spikes on an individual particle.

In the present study, the broadly cross-reactive, non-neutralizing F240 epitope (37) in the “Cluster I” domain of gp41 served as the focal point for our experiments. The F240 epitope is located within the immunodominant disulfide loop region of gp41 (38, 39), which is commonly immunoreactive on the surfaces of free virions (33, 34, 36, 40, 41). Although the F240 epitope is occluded on intact Env trimers, it seems to be exposed on undefined Env structures in which gp41 is oxidized (41). Passive transfer of mAb F240 exhibited a marginal degree of protective efficacy against SHIV challenge in macaques (42); a mAb against a related Cluster I epitope, 246D, mediated protection against HIV-1 infection in humanized mice (31); another related mAb, 7B2, (41) reduced the number of transmitted/founder SHIV variants in passively immunized macaques (43). Here we report that the antibodies directed against the F240 epitope and neutralizing gp120 epitopes bind concurrently to single virions via a shared Env-derived structure. Further, our data suggest that most particles in a virus population harbor such structures.

## Materials and Methods

### HIV-1 Pseudovirus Production

HIV-1 BaL and HIV-1 JRFL pseudoviruses were generated by co-transfection of HEK293T cells with an Env-deficient HIV-1 backbone plasmid pNL4-3-ΔE-EGFP along with Env-expression plasmids (44, 45) pHIV-1-BaL 0.1 (obtained through the AIDS Research and Reference Reagent Program, Division of AIDS, NIAID) and pCAGGS-JRFL (kindly provided by J. Binley, Torrey Pines Institute of Molecular Studies, San Diego, CA). Transfections were accomplished using FuGENE 6 (Roche, Indianapolis, IN) transfection reagent at a 3:1 reagent-DNA ratio. To produce the infectious molecular clone of transmitted/founder (T/F) HIV-1 AD17 virus (46), HEK293T cells were transfected with the AD17 plasmid (kindly provided by B. Hahn, University of Pennsylvania) at a FuGENE-to-DNA ratio of 3:1. Virions-containing supernatant was harvested after 3 days, and concentrated about 10-fold by incubating with PEG-*it*™ virus precipitation solution (System Biosciences, Mountain View, CA) for 18 h at 4°C as recommended by vendor. The antigen content of all virion preparations was quantified using p24 and gp120 antigen capture ELISAs. Infectivity was established using standardized procedures (47) and quantified as function of TCID_50_ in TZM-bl cells. HIV-1 BaL and HIV-1 JRFL pseudoviruses with gp120 to p24 ratio of 1:10–1:50, and 200,000–500,000 TCID50/mL; HIV-1 AD17 T/F with gp120 to p24 ratio of 1:200, and 600,000 to 1,000,000 TCID50/mL were used for FCS measurements. The Aldrithiol-2 (AT-2) inactivated (48, 49) HIV-1 BaL virus produced in SupT1-CCR5 CL.30 cells was generously provided by Dr. Jeff Lifson (AIDS Vaccine Program/NCI, Frederick, MD). pEGFP-Vpr (cat# 11386 from Dr. Warner C. Greene) plasmid (50) obtained through the AIDS Research and Reference Reagent Program, Division of AIDS, NIAID, NIH, was used to generate fluorescent HIV-1 BaL pseudoviruses.

### Antibodies

mAbs b12, 2G12, PG9, and F240 were purchased from Polymun Scientific (Vienna, Austria). mAbs 17b and N49P7 (14) were expressed from plasmid clones in HEK293T cells using an IgG1 backbone for heavy-chain variable regions and either a κ- or λ-chain expression vector for light-chain variable regions. mAbs were purified from culture supernatants by protein-A chromatography. Fabs of b12, 2G12, PG9, N49P7 or F240 were prepared from purified IgG (10 mg/ml) by proteolytic digestion with immobilized papain (Pierce, Rockford, IL) and purified using protein A (GE Healthcare, Piscataway, NJ), followed by gel filtration chromatography on a Superdex 200 16/60 column (GE Healthcare, Piscataway, NJ). All mAbs or Fabs were fluorescently labeled and purified with Alexa 488, 568, or 647 monoclonal Antibody Labeling Kit (Invitrogen, Molecular Probes, Eugene, OR). Briefly, the Alexa dye has a succinimidyl ester moiety that reacts efficiently with primary amines of antibody to form stable dye-protein conjugates. Each labeling reaction was performed with 100 μg of a mAb or Fab. The labeled antibody was separated from unreacted dye by centrifugation through a spin column at 1,100x g for 5 min. Recovered antibodies were dialyzed against phosphate buffered saline as necessary. Labeled mAbs or Fabs were quantified by a UV-vis spectrometer (Nanodrop 2000, Thermo-Scientific, Wilmington, DE). Dye to protein ratios were determined by measuring absorbance at 280 nm (protein) vs. absorbance at corresponding wavelength for Alexa 488, 568, or 647. Conjugated mAbs or Fabs used in our experiments had an optimal dye to protein ratio in the range of 1–2 of dye per molecule of mAb or Fab.

### Two-Color Fluorescence Correlation Spectroscopy

Fluorescence Correlation Spectroscopy (FCS) is a methodology that allows real-time detection of multiple protein-protein interactions in solution, by measuring diffusion and reaction kinetics of fluorescently-labeled biomolecules (51). Two-color FCS (52) has been used to monitor two different mAbs bound to HIV-1 virions. The binding of Alexa 488 or 647-labeled mAbs b12, 2G12, 17b, F240, and anti-RSV antibody, Synagis (negative control) to HIV-1 virions was monitored by tracking diffusion of their fluorescent label across the observation area, where unbound antibodies will diffuse much faster than those that bound viral particles as described in Ray et al. (36). Briefly, HIV-1 BaL pseudovirions were diluted to 10 μg/mL p24 equivalent in a 100-μL reaction volume (gp120:p24 ratio of 1:50), and were first incubated with 100 μg/mL non-specific IgG1 (1.5 μL of a 7 mg/mL stock) for 90 min at 37°C to block non-specific binding. Then 1 μL of the test Alexa conjugated mAbs (2 μg/mL of each mAb) was introduced and allowed to interact with pseudovirions for 90 min at 37°C. For spectroscopic measurements, 11 μL of the reaction mixture was loaded onto an FCS slide reservoir, sealed, then placed on a time-resolved confocal microscope (ISS Q2) with a high numerical aperture (NA = 1.2) water objective (60x magnification). The excitation source was a Fianium SC-400 super-continuum laser. NKT super-select acousto-optical tunable filter (AOTF) filter was used to select the excitation wavelengths. The beam after AOTF was passed through narrow bandpass clean-up filters. The samples were excited with two coincident excitation at 470 and 635 nm, and fluorescence signals from the Alexa 488 (A488) or Alexa 647 (A647) mAbs were collected in two separate detection channels in the 500–550 nm and 650–720 nm region over 60 s in a constant detection volume (~1 fL) that is continuously replenished. ISS Vista vision software was used to generate the autocorrelation function of the fluorescent fluctuations of the Alexa labeled mAbs. The autocorrelation function of fluorescence intensities is given by the product of the mAb intensity at time *t, I(t)* with the intensity after a delay time τ, *I(t*+τ*)*, typically in the range from 10^−2^ to 10^2^ ms, averaged over the 60 s of measurement.

For experiments in which only the conjugated mAbs and no virions were present, the autocorrelation function was fitted with a single species diffusion model equation. Diffusion coefficients of the fluorescent species under these conditions were routinely determined to be 60 μm^2^/s. These values matched what was predicted for 150 kD IgG molecules in solution. In reactions with mAbs and virions, the autocorrelation was fit to a two-species diffusion model. In this operation, one species had the unbound mAb diffusion coefficient of 60 μm^2^/s, and the second a diffusion coefficient of 6 μm^2^/s, matching the predicted behavior of fluorescent mAbs bound to a 100 nm retroviral particle. The fitting equations were also used to determine the percentage of total mAb exhibiting the slower diffusion rate in reactions with virions. The mathematical derivation and application of the equations is described in (36). The cross-correlation measures (52, 53) between the two separate detection channels determined if two signal intensities were correlated; i.e., fluctuated in concert or independently. Only pairs of co-incident photon counts from two distinct channels (500–550 and 650–720 nm in the present case) will show positive correlation amplitude. Only dual-color cross-correlation data of two different antibodies in the presence of HIV-1 virion showing positive correlation amplitude were taken as evidence that two different antibodies bound to the same virion particle.

### Characterization of Single Virion FCS Measures

As dual-label FRET measures cannot distinguish the number of targets based on signal intensity, they are most clearly interpreted when there are limiting amount of virions in the focal volume. This situation can be favored by appropriate dilutions of the virus stock. In theory, the amount of p24 in a virus preparation may be used for this purpose, assuming 10^4^ virus particles per picogram of p24 (54, 55). To better determine how p24 measures could be used in this manner with our virus preparations, we generated HIV-1 BaL pseudoviruses using the usual methods, but also containing an eGFP.Vpr fluorescent marker. The eGFP.Vpr HIV-1 BaL stock contained 3 μg/ml of p24, which based on 10^4^ virus particles/picogram of p24 (54, 55), converts to 3 × 10^7^ particles/μL or roughly 0.03 virus particle per femtoliter assuming that virions are evenly dispersed. Serially dilutions of virus were then analyzed by FCS (Figure S1), which detected the eGFP.Vpr signal and thereby quantified the number of particles in the focal volume at any one time. The number of fluorescent virions were determined by fitting the autocorrelation plot and extracting the correlation amplitude G(0) (51, 53). The number of molecules detected in the FCS focal volume is inverse of the correlation amplitude (51, 53). The diffusion coefficient of the eGFP.Vpr HIV-1 BaL pseudoviruses signals (5 μm^2^/s) matched those previously determined for single pseudovirus particles (36). As shown in Figure S1, there was the expected linear relationship between p24 concentration and number of virions measured by FCS. Notably, the FCS analyses detected substantially more virions in the focal volume than what was predicted by p24 measures (for example, although 3 μg/ml was predicted to translate into 1 virion/33fL; FCS indicated there was 1 virion/1.6 fL). FRET-FCS can only be performed with unlabeled virions as the signal from the eGFP fluorescent virion will interfere with the signal from fluorescent tagged mAbs or Fabs. Thus, a conversion factor was determined and used for experiments with other unlabeled viruses (see below) to normalize p24 concentrations to the probable numbers of virions (~1.5) being seen in the FCS focal volume of 1fL.

### FRET-FCS Measurements

For FRET measurements, the Fabs (b12, 2G12, PG9, N49P7 or F240) were labeled with either donor (Alexa 488) or acceptor (Alexa 568) probes (Invitrogen mAb labeling kit). Dye-to-protein ratios were determined by measuring absorbance at 280 nm (protein) vs. 488 or 577 nm (dye). The dye-to-protein ratios were between 1 and 2. We specifically aimed to keep this low label of dye labeling as we are using a single molecule fluorescence method and minimally perturb the functionality of the protein. FRET measurements were performed in a confocal microscope (ISS Q2) equipped with a supercontinuum laser and AOTF in order to excite the molecules during its diffusion through the confocal volume. ISS Vistavision software was used to generate the FRET histogram and further analyses. FRET measurements were performed after forming complex with the HIV-1 BaL, HIV-1 JRFL, AT-2 inactivated HIV-1 BaL or HIV-1 AD17 T/F virions with donor-labeled Fab and acceptor labeled Fab. The number of virions at any given time was around 1.5 particle in the 1fL focal volume as described above.

Fluorescence responses from the donor and the acceptor molecules were separated by a dichroic beam splitter and detected by two avalanche photodiode detectors (APD) using the method of time-correlated single photon counting and the Time-Tagged Time-Resolved (TTTR) mode of the Becker and Hickl SPC-150 module. High quality bandpass (Chroma) filters were used for laser clean-up and recording donor and acceptor fluorescence in two separate detection channels. The collected single photon data was binned by 3.4 ms (corresponding to the diffusion time of the 100 nm diameter virion particle) in each channel (donor or acceptor), which resulted in intensity-time traces recorded for 120 s. Threshold values in each channel were used to identify the single molecule bursts from the corresponding background signal level. Fluorescence bursts were recorded simultaneously in donor and acceptor channels and FRET efficiencies were calculated using *E* = I_*A*_/(I_*A*_ + γI_*D*_) where I_*D*_ and I_*A*_ are the sums of donor counts and acceptor counts for each burst, taking into account the possible difference in the detection efficiency (γ) in two separate channels (56–60). The analyses revealed fractional quantity of FRET efficiency events for a specified bin and recorded time of the donor-acceptor intensity traces. For a measurement time of 120 s and sampling frequency of 300, total number of 36,000 events can be possibly obtained. It is important to note that an event is likely no more than two virions in the FCS observation volume of 1fL based on input concentration of p24 as shown in Figure S1. For each sample containing donor Fabs, acceptor Fabs and HIV-1 virions, fractions of FRET events relating to the total possible events for a given bin time or sampling frequency and measurement time were determined and subsequently the number of occurrences vs. FRET efficiency histogram plots were generated. The donor-to-acceptor distance (*r*) in terms of efficiency of energy transfer (*E*) and Förster Distance *(*R_0_) is given by *r* = R_0_ *[1/E – 1]*^1/6^. We have used the value of R_0_ of 6.2 nm for the Alexa 488 (donor) and Alexa 568 (acceptor) pair for estimating the donor-to-acceptor distances. In addition to FRET measurements we have also performed FCS measurements to assess the *in vitro* binding of Fab fragments to HIV-1 virions. Consequently, we determined the translational diffusion coefficients of Alexa 488 or 568 labeled Fabs and the corresponding bound virion complexes from FCS measurements. The FCS measurements and analyses were performed as previously reported (21, 36, 57–60).

### Assembly of Structural Models of b12 and 2G12 Bound to HIV Env

The model was assembled based on the available CryoEM structure of the virion associated HIV-1 trimer complexed with b12 Fab [PDB: 3DNL, (61)] and crystallographic structure of 2G12 Fab bound to Man_9_GlcNAc_2_ [PDB code: 6N2X, (62)]. 2G12 Fab was modeled into the b12 Fab-HIV-1 trimer by superimposition of the Man_9_GlcNAc_2_ moiety of the 2G12 Fab- Man_9_GlcNAc_2_ complex to the trimer at N-linked glycan at position 332 (62). The distances are measured from the center of each variable domain of Fab.

## Results

Previously we used FCS and fluorescent labeled proteins to examine the binding of individual anti-envelope mAbs or sCD4 to HIV-1 particles representing various strains with all reactants in solution (21, 36, 41). These studies showed that the Alexa -labeled anti-gp120 bNAbs 2G12 (63) and b12 (64), and the non-neutralizing anti-gp41 mAb F240 (37, 41), bound efficiently and consistently to virions (21, 36). However, these studies did not address whether two antibodies, each of different specificity, bind to the same virion or to the same Env structure on a particle surface. We reasoned that dual color detection and FRET-FCS should afford a means to address this question.

### Epitope Exposure on Single Virions by Dual Color FCS

**Figure 1:**
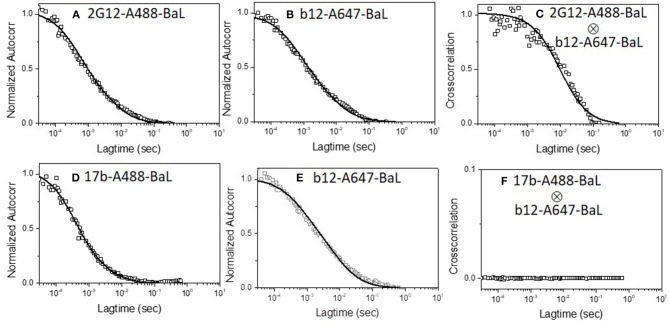
Dual-color correlation curves of **(A–C)** 2G12-A488 and b12-A647 and **(D–F)** 17b-A488 and b12-A647 with HIV-1 BaL virions. **(A,D)** show autocorrelation plots of Alexa 488 labeled mAbs. **(B,E)** show autocorrelation plots of Alexa 647 labeled mAbs. **(C,F)** show cross-correlation curves of b12-A647 and 2G12-A488 or b12-A647 and 17b-A488 with HIV-1 BaL virions. Two color excitation wavelengths at 470 and 635 nm were used. All experiments were repeated three times with similar results.

We first applied the dual color detection method to explore the binding of two different mAbs to single HIV-1 BaL pseudovirus particles. We employed anti-envelope mAbs including b12 [a broadly neutralizing CD4 binding site antibody (64)], 2G12 [against a carbohydrate cluster on gp120 (63)], and F240 [against a cluster 1 epitope in gp41 (37, 41)] labeled with either Alexa 488 or Alexa 647. Monoclonal antibody 17b was tested as a negative control. This mAb recognizes a CD4-induced epitope on gp120 (65), binds weakly to HIV-1 BaL in the absence of sCD4, and partially competes with b12 for gp120 binding due to partial epitope overlap (20, 66). Thus, mAbs 17b and b12 are unlikely to bind the same virion except through non-specific processes. Figure 1 shows the dual-color FCS measurements of Alexa-488 labeled 2G12 and Alexa-647 labeled b12 binding. Autocorrelation plots (Figures 1A,B) showed that in the reaction 42 and 45% of b12 or 2G12 mAbs, respectively, adopted the slower diffusion coefficient (6 μm^2^/s) marking virion-bound mAb. Similar binding efficiencies for these mAbs were reported previously (36). Importantly, cross-correlation analyses (51, 53) (Figure 1C) of signals simultaneously detected in the two channels could also be fitted to the same single diffusion coefficient 6 μm^2^/s. Such findings reflect that both 2G12 and b12 being bound to the same object, having the size of a retrovirus particle. In comparison, analyses of b12-A647 and 17b-A488 mixed with HIV-1 BaL virions showed no cross-correlation in binding signals (Figures 1D–F). Taken together, the data obtained with mAb pairs 2G12 and b12 indicated that the dual color coincidence FCS assay system could reflect the epitope exposure patterns on virus particles in solution.

**Figure 2:**
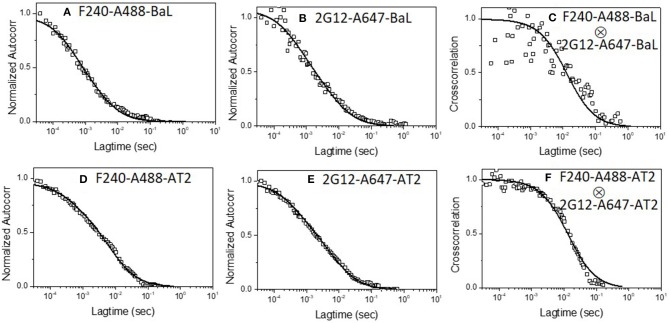
Dual-color correlation curves of F240-A488 and 2G12-A647 with **(A–C)** HIV-1 BaL, and **(D–F)** AT-2 HIV-1 BaL virions. **(A,D)** show autocorrelation plots for F240-A488 with HIV-1 BaL and AT-2 HIV-1 BaL virions; **(B,E)** show autocorrelation plots of 2G12-A647 with HIV-1 BaL, and AT-2 HIV-1 BaL virions; **(C,F)** show cross-correlation curves of F240-A488 and 2G12-A647 with HIV-1 BaL and AT-2 HIV-1 BaL virions. The experiment was repeated three times with similar results.

A second set of experiments examined whether a single virion binds 2G12 neutralizing mAbs along with mAb F240. In these experiments, mAb F240 was labeled with Alexa-488 and mAb 2G12 with Alexa-647, the targets were again HIV-1 BaL pseudoviruses. Figure 2 shows the dual-color FCS measurements of Alexa-488 labeled F240 and Alexa-647 labeled 2G12 binding to HIV-1 BaL virions. Autocorrelation plots (Figures 2A,B) showed that in the reaction 35 and 45% of F240 or 2G12 mAbs, respectively, adopted the slower diffusion coefficient (6 μm^2^/s) marking virion-bound mAb. Similar binding profiles of fluorescently labeled F240 or 2G12 to HIV-1 BaL were reported previously (36). As in the experiments pairing 2G12 with b12 (Figure 1), cross-correlation analyses showed simultaneous signal detection in the two channels (Figure 2C) fitting a single diffusion coefficient of ~6 μm^2^/s, indicating that 2G12 and F240 bound to the same virion particle. To verify these results further, the binding of both 2G12 and F240 mAbs was assessed with AT-2 inactivated HIV-1 BaL virions produced and purified in a different manner (41, 49). Similar auto-correlation (Figures 2D,E) and cross-correlation (Figure 2F) profiles were observed with Alexa-488 labeled F240 and Alexa-647 labeled 2G12 binding to AT-2 inactivated HIV-1 BaL virions.

### Epitope Exposure on Individual Env Structures on HIV-1 Virions by FRET-FCS

Having determined that the above mAb pairs concurrently bind single virus particles, we next examined whether combinations of the above antibodies were attaching to the same Env structure on the virion. The FRET-FCS method relies on standard dipole-dipole interactions between paired donor and acceptor fluorophores, which occurs within the distance range of 2–10 nm. Thus, paired fluorescent mAbs will not create FRET signals unless they bind to tightly localized epitopes. To mitigate spatial differences caused by the probe length (i.e., whole IgG) and more precisely estimate the donor-acceptor distances, the FRET-FCS experiments employed labeled Fab fragments. The use of Fabs also enabled interpretations of FRET data vs. available structural data for Fab-Env complexes. The fluorophores pairs used in all experiments were Alexa-488 (A488 donor) and Alexa-568 (A568 acceptor). The test viruses (see below) were used at final concentrations of 10 μg/ml, which, based on relationship shown in Figure S1, was predicted to produce roughly 1.5 virions in the focal volume at any time.

**Figure 3:**
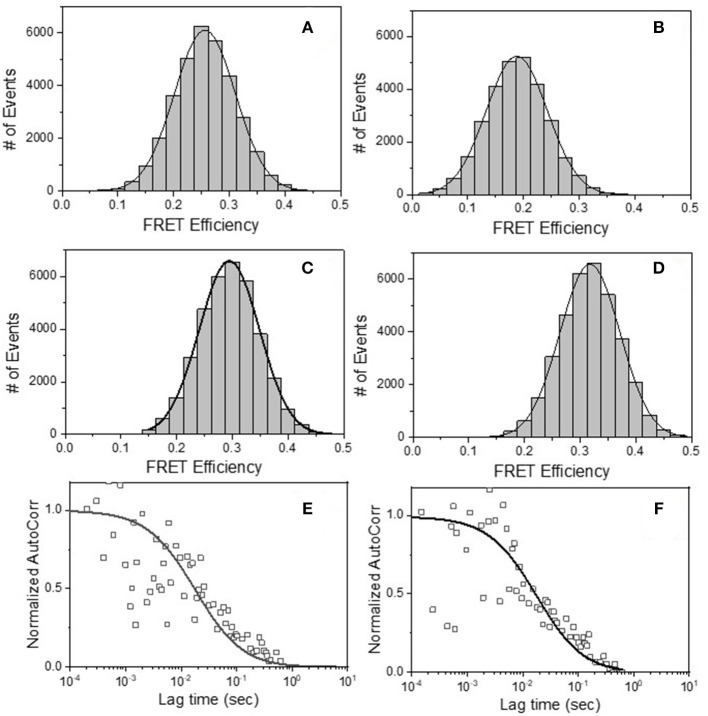
FRET histograms of **(A)** b12 Fab-A488 and 2G12 Fab-A568 **(B)** 2G12 Fab-A488 and b12 Fab-A568, **(C)** b12 Fab-A488 and F240 Fab-A568 and **(D)** F240 Fab-A488 and b12 Fab-A568 with HIV-1 BaL virions. The solid lines in **(A–D)** are fit with a Gaussian distribution to the experimental FRET histogram data. Autocorrelation plots of the acceptor channel for **(E)** b12 Fab-A488 and 2G12 Fab-A568 and **(F)** b12 Fab-A488 and F240 Fab-A568 with HIV-1 BaL virions. The solid lines in **(E,F)** represent the fit to the experimental data. All experiments were repeated three times with similar results.

**Figure 4:**
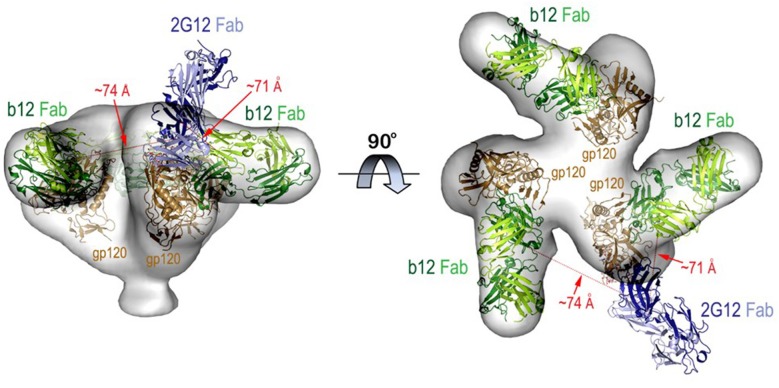
Molecular model for b12 and 2G12 Fabs bound to the same HIV-1 trimer. The model was built using cryoEM structure of the virion associated HIV-1 trimer complexed with b12 Fab and 2G12 Fab-Man_9_GlcNAc_2_ complex as described in Methods. The relative distances (shown as red dotted lines) between the Fabs were measured from the centers of variable domains of each Fab. Molecular surface is displayed over the HIV-trimer and Fabs are shown in ribbon diagrams.

The FRET-FCS system was first applied toward analyses of neutralizing epitopes. Reaction mixtures were constructed with fluorescent labeled 2G12 (A488 donor) and b12 (A568 acceptor) Fabs and HIV-1 BaL virions (see methods). As shown in Figure 3, FRET signals from the acceptor probe were indeed detected, following a diffusion coefficient of 6 μm^2^/s clearly distinguished from the ~80 μm^2^/s value expected for an unbound Fab. The FRET signals fit a Gaussian profile with a mean FRET efficiency of ~25% (Figure 3A). These data indicated that the two Fabs occupied a highly localized space on bound virions. FRET efficiency was not biased by the donor/acceptor labeling configuration as swapping the donor-acceptor labeling between b12 Fabs and 2G12 Fabs yielded highly similar measures (Figure 3B). According to Forster's equation (see methods) the calculated mean FRET efficiencies were consistent with a situation where the donor and acceptor Fabs bound to virions at an average distance of 7.4 nm apart. To validate these findings, we used existing structural information (61, 67) to model (see methods) the binding of 2G12 and b12 Fabs to a single HIV-1 trimer (Figure 4). This exercise predicts that the Fabs would be spaced ~7.1 nm apart if bound to the same protomer and ~7.4 nm apart if each is bound to a different protomer in the same trimer. In either case, the modeling predictions closely match those calculated from the FRET efficiencies. However, it must be noted that the Alexa conjugation chemistry does not place fluorophores at single, specified positions along the length of Fabs. Thus, FRET efficiencies, and derivative donor-acceptor distances, reflect aggregate measures of spatial ranges between paratopes and fluorophores on the two Fab probes. Given that Fabs are ~3 nm long, up to 3 nm in extra distance between epitopes could be added when fluorophores are positioned at maximum distances from paratopes on each probe. However, even in this extreme situation the two epitopes can still be placed within the spatial constraints of a single trimer.

The prevalence of the above associations in the virus population was considered by comparing the events that exhibited any degree of FRET signal (Figures 3A,B) to the total number of events possibly observed under the reaction conditions used (see methods for calculation). These analyses indicated that in reactions with b12 Fab-A488 and 2G12 Fab-A568 or 2G12 Fab-A488 and b12 Fab-A568, donor-acceptor pairs, FRET signals covered 70–75% of the total possible observable events under the experimental system. Taken together, the data indicated that b12 and 2G12 Fabs bound to a single Env structure presented by the majority of particles observed in the FCS system.

We next applied FRET-FCS to reaction mixtures containing Fab fragments of b12 and F240 mixed with the HIV-1 BaL virions. The labeling of the two Fabs (A488 donor vs. A568 acceptor) were reciprocally interchanged to confirm that any FRET measures were not biased by the donor/acceptor labeling strategy. Moreover, FRET signals were not detected in reactions with donor (A488) and acceptor (A568) labeled Fabs in the absence of HIV-1 virions (data not shown). This was expected, as there was no reason for the Fabs to remain in association outside of random diffusion such that a FRET signal could be generated. As shown in Figure 3, FRET signals were again detected when combinations of b12 Fab-A488 and F240 Fab-A568 (Panel C) or F240 Fab-A488 and b12 Fab-A568 (Panel D) were mixed with HIV-1 BaL. Single diffusion coefficients calculated for the acceptor channel were 6 μm^2^/s, consistent with signals emanating from objects with the size of HIV virions (Figures 3E,F). As above, the number of events that exhibited any degree of FRET signal (Figures 3C,D) were compared to the total number of events possibly observed in the system considering the recording time and sampling frequency (see methods). The analyses showed that in reactions with b12 Fab-A488 and F240 Fab-A568 or F240 Fab-A488 and b12 Fab-A568 donor-acceptor pairs, FRET signals covered 75 and 72% of the total possible observable events in the system. Gaussian fitting to the FRET histogram data (Figures 3C,D) reflected FRET efficiencies of 30–35% regardless of dye pairing. This in turn indicates that b12 Fab and F240 Fab bind in close proximity; ~7.1 nm apart (in the range of 6.6–8 nm) on some sort of gp120-gp41 complex.

**Figure 5:**
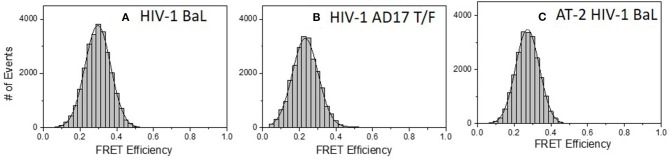
FRET histograms of 2G12 Fab-A488 and F240 Fab-A568 reacted with different virus types: **(A)** HIV-1 BaL, **(B)** HIV-1 AD17 transmitted/founder, and **(C)** AT-2 HIV-1 BaL. The solid lines in **(A–C)** are fit with a Gaussian distribution to the experimental FRET histogram data. All measurements were repeated three times with similar results.

Similar experiments were carried out with 2G12 Fab-A488 and F240 Fab-A568 pairs. Figure 5A shows the FRET histogram of the Fabs with HIV-1 BaL virions. Again, the histogram plot reflected 30% FRET efficiency and Fab positions ~7.1 nm apart (in the range of 6.4–8.1 nm). The FCS auto-correlation curve in the acceptor channel exhibited only a single diffusion corresponding to a HIV-1 BaL virion. To determine whether the FRET-FCS measures with HIV-1 BaL virions were generalizable, additional experiments were conducted in which the targets were either AT-2 inactivated HIV-1 BaL virus (produced in SupT1-CCR5 CL.30 cells) (41, 49) or a transmitted founder (T/F) infectious molecular clone (AD17) produced in HEK293T cells (36). As shown in Figure 5, both viruses exhibited FRET signals when reacted with 2G12 Fab-A488 and F240-Fab-A568 donor-acceptor pairs. The FRET pattern with the AT-2 inactivated HIV-1 BaL (Figure 5C), and predicted binding distance of the Fabs were comparable to what was detected with the HIV-1 BaL pseudovirus (Figure 5A). Between the two viruses, a slightly lower FRET efficiency was observed for HIV-1 AD17 (Figure 5B). One possible explanation is that HIV-1 BaL and AD17 present slightly different gp120-gp41 surface structures coincidently reactive with both Fab F240 and Fab 2G12. In any case, FRET-linked concurrent binding of the Fabs comprised 66, 58, and 58% of the total possible events in the system (see methods) for the HIV-1 BaL; HIV-1 AD17 or AT-2 inactivated HIV-1 BaL virions, respectively. These FRET-reactive fractions of the virus population were lower than what was observed with co-localized binding of b12 and F240 Fabs (see above).

**Figure 6:**
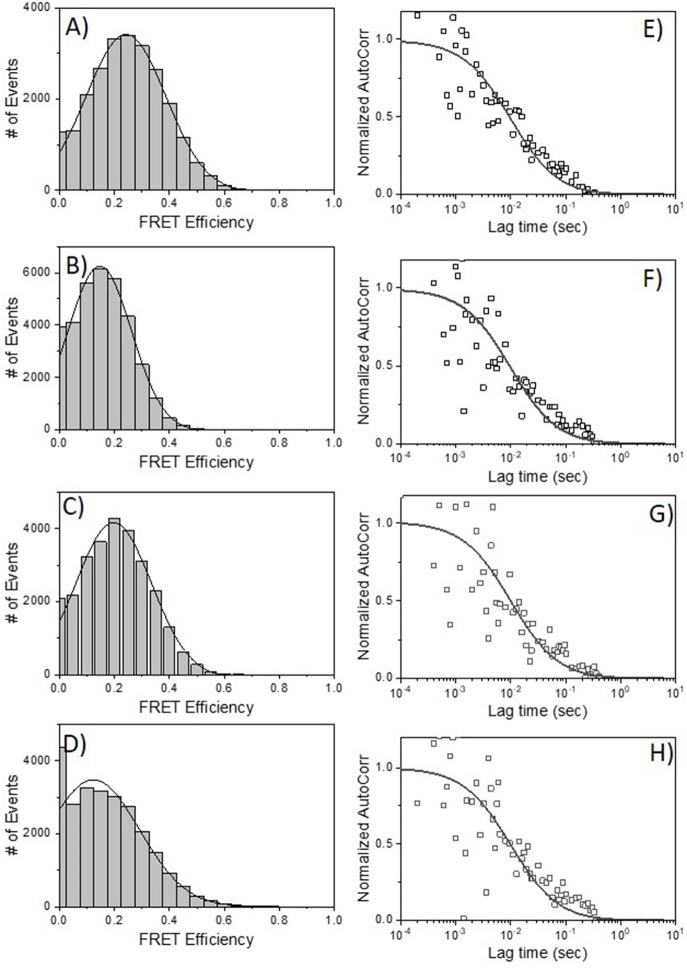
FRET histograms of Fab pairs tested with Tier 2 HIV-1 JRFL virions. **(A)** 2G12 Fab-A488 and F240 Fab-A568; **(B)** b12 Fab-A488 and F240 Fab-A568; **(C)** N49P7 Fab-A488 and F240 Fab-A568 and **(D)** PG9 Fab-A488 and F240 Fab-A568. The solid lines in **(A–D)** are fit with a Gaussian distribution to the experimental FRET histogram data. Autocorrelation plots of the acceptor channel for **(E)** 2G12 Fab-A488 and F240 Fab-A568; **(F)** b12 Fab-A488 and F240 Fab-A568; **(G)** N49P7 Fab-A488 and F240 Fab-A568 and **(H)** PG9 Fab-A488 and F240 Fab-A568 with HIV-1 JRFL virions. The solid lines in **(E–H)** represent the fit to the experimental data. All measurements were repeated three times with similar results.

HIV-1 BaL is a “tier 1b” virus, meaning it has a relatively “open” Env structure more sensitive to neutralization by a wider variety of anti-Env mAbs. Thus, we performed additional FRET-FCS experiments with a tier-2, CCR5 tropic JRFL pseudovirus; i.e., one expressing a “closed” Env structure resistant to neutralization by most mAbs. Reactions were run with fluorescent labeled neutralizing Fabs of 2G12, b12, N49P7 [a CD4 binding site potent broadly neutralizing mAb (14)] and PG9 [a potent broadly neutralizing mAb targeted to quaternary structure in the V1-V2 loops of gp120 (68–70)] along with F240 Fab. The PG9 gp120 epitope is predicted to be at a substantial distance (~9 nm) from F240 in a single trimer, approaching the limit where FRET becomes undetectable. Nevertheless, PG9 Fab was tested as it interacts with two gp120 protomers in the trimer (68–70) and thus could inform the nature of the target antigen. Specifically, any FRET between PG9 and F240 Fab pairs could indicate the presence of two gp120s in the cognate Env structure. As shown in Figure 6, FRET signals were detected when combinations of 2G12 Fab-A488 and F240 Fab-A568 (Panel A); b12 Fab-A488 and F240 Fab-A568 (Panel B) were reacted with HIV-1 JRFL. The average FRET efficiencies for the 2G12-F240 and b12-F240 combinations were 25 and 20%, respectively similar to those observed with the tier-1 HIV-1 BaL virions. Accordingly, the calculated average distances for the 2G12-F240 and b12-F240 Fab pair combinations with HIV-1 BaL virion were 7.4 and 7.8 nm, respectively. FRET signals for the 2G12-F240 and b12-F240 Fab pairs with HIV-1 JRFL virions comprised 54 and 61% of the total possible observable events in the system.

FRET signals and efficiencies of 20% between N49P7 Fab-A488 and F240 Fab-A568 with HIV-1 JRFL virions (Figure 6C) reflected an average distance of 7.8 nm (in the range of 6.6–8.6 nm) between the two Fabs. This distance is similar to the average distance observed with b12-F240 Fab pairs, as expected, considering both b12 and N49P7 are CD4bs mAbs. FRET signals for the N49P7-F240 Fabs with HIV-1 JRFL virions comprised 56% of the total possible observable events in the system. The histogram for the combinations of PG9 Fab-A488 and F240 Fab-A568 mixed with HIV-1 JRFL (Figure 6D) reflected a FRET efficiency of about 10% for PG9-F240 pairs suggesting an average distance of 9 nm (in the range of 7.5–9.5 nm) between the two Fabs. As noted above, this result is in accordance with the available structural information of the distance between the PG9 epitope in the V1-V2 region of gp120 and F240 epitope in gp41 (41, 68–70). Notably, FRET signals from the PG9-F240 Fabs with HIV-1 JRFL virions comprised only 32% of the total possible observable events. The fit to autocorrelation plots (Figures 6E–H) for the acceptor channel for all the neutralizing and non-neutralizing Fab combinations showed single diffusion coefficients of 6 μm^2^/s, consistent with signals emanating from objects with the size of HIV-1 virions.

## Discussion

Our previous FCS experiments showed that neutralizing mAbs 2G12, b12, and PG9; and the non-neutralizing anti-gp41mAb F240, bind often and efficiently to various pseudoviruses and full length infectious molecular clones produced in different ways (36). However, those analyses did not distinguish whether each mAb bound a specific subset of virions, nor did they reflect how individual virions react with multiple mAb specificities. Under the FCS conditions used here, we were able to make such determinations as each measured fluorescence event stemmed from roughly 1–2 particles in the focal volume being assayed (Figure S1). The diffusion coefficients of the collected signals further affirmed that they arose from objects the size of retroviral particles.

Dual color FCS established that the neutralizing mAbs 2G12 and b12 concurrently bound the majority of individual HIV-1 BaL pseudovirions in the population (Figure 1), and frequently enabled FRET signals (Figure 3) indicating occupancy of the same functional trimer, consistent with structural predictions obtained by *in silico* modeling (Figure 4). Observations of neutralizing antibodies bind concurrently to the same trimer are not particularly surprising from a virological standpoint; but the data as such support the utility of the approach. Moving to FRET analyses of other epitopes, a more unexpected yet consistent observation was the concurrent and highly localized binding of F240 Fab and 2G12 Fab to HIV-1 BaL and JRFL viruses; AT-2 inactivated HIV-1 BaL virions from another source; and the HIV-1 AD17 T/F molecular clone (Figures 5, 6). We also detected tightly localized binding of F240 Fab and N49P7 or PG9 Fab on the Tier 2 HIV-1 JRFL pseudoviruses (Figure 6).

**Figure 7:**
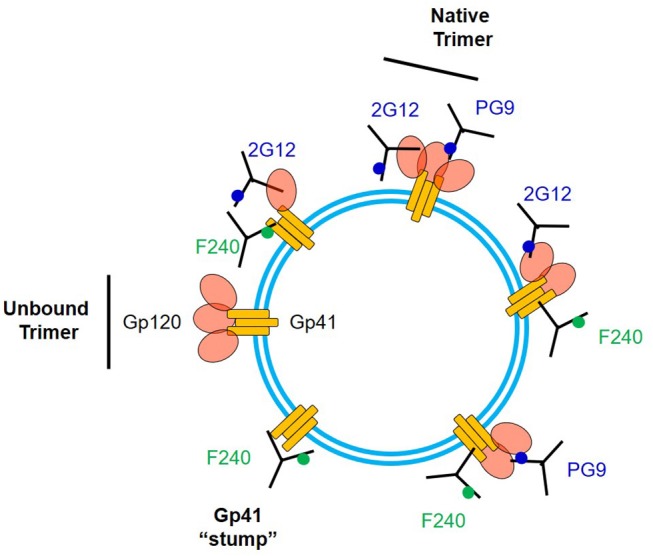
Array of some possible structures on virion surfaces including ones that could co-express F240 and neutralizing epitopes. 2G12 and PG9 epitopes are shown as examples of the latter.

The F240 epitope is occluded on intact Env trimers (41) and is often assumed to reside only on gp41 “stumps” on the virion surface (26). We cannot eliminate the possibility that some virions present such gp41 structures. Nevertheless, our data suggest the existence of another, previously unexpected type of Env structure that expresses the F240 epitope within 10 nm of a variety of broadly neutralizing sites, spaced to avoid steric competition between cognate Fabs. It seems implausible that multiple Env-derived structures (e.g., a gp41 “stump” and non-specifically adsorbed gp120 monomers of some sort) could develop a surface structure comprising the epitope positioning we measured. A more likely possibility is that the F240 and gp120 epitopes are co-located on a misfolded or partially denatured trimer segment that remains membrane-anchored, retains one or more gp120 protomers, and exposes the gp41 Cluster I domain. Detection of FRET with F240 and PG9 Fabs suggests the presence of two closely spaced gp120 protomers in the target antigen. However, FRET signals for these Fabs were detected less often (roughly 30%) among the total possible observable events, compared to the other epitope pairs. One explanation for the difference is that F240 and PG9 are co-expressed on a relatively infrequent structure, compared to a more common one expressing F240 with 2G12, b12, or N49P7. Another explanation could be dynamic and heterogeneous positioning of PG9 Fab vs. F240 Fab, sometimes outside the FRET window, within the virion population. Figure 7 summarizes potential scenarios for F240 and neutralizing anti-gp120 epitope presentation on virions that are consistent with the FCS data considered above. It must be noted that these sorts of Env antigens have not been apparent via virion capture approaches. One possible explanation is that the gp120-gp41 interactions in these structures are too fragile to withstand the capture process without experiencing further degradation.

Humoral immunity includes multiple Fab- and/or Fc-driven mechanisms capable of suppressing viral infections. Thus, any conserved viral epitopes on replication-competent viruses, even if non-neutralizing, are potential points of vulnerability. Humoral responses against influenza and Ebola viruses are important cases in point, where protection from infection by non-neutralizing antibodies has been repeatedly demonstrated (71–75). In the HIV-1 system, non-neutralizing epitopes are often considered in the context of aberrant Env structures (e.g., gp41 “stumps”) on replication-defective particles while broadly neutralizing epitopes are often taken as a marker for replication-competent virions (9, 25, 26, 33, 34). However, our investigation of unadulterated, single virions in solution reveals that there is overlapping expression of both sorts of epitopes on the same virion and even on the same surface Env structures. Further, this overlap can be apparent on the majority of virions in a population. Such data point toward the potential value of developing vaccines that elicit polyclonal anti-Env responses comprising both neutralizing and non-neutralizing antibodies, which in concert may guide multiple effector mechanisms to viruses or virus infected cells.

Going a step further, our data provide a virological basis for considering whether and how certain non-neutralizing responses alone may effectively block infection, by vaccination or other preventive measure targeting gp41. In various animal models of HIV-1 infection, antibodies to F240 and related/overlapping Cluster I epitopes on gp41 have already been linked to varying degrees of resistance (31, 42, 43, 76, 77). Our data indicate that such *in vivo* effects may involve selective activity against virions, particularly given other evidence that Cluster I epitopes are poorly expressed on infected cells (41). Vaccines that generate such responses, particularly at points of mucosal exposure, merit exploration and may now be developed using new information concerning Env structures on free virions.

## Data Availability

All data sets generated for this study are included in the manuscript and/or the Supplementary File.

## Author Contributions

KR designed the research, performed experiments, analyzed the data, and wrote the manuscript. MM and CO performed experiments. MP performed structural modeling. GL analyzed the data. AD designed the research, analyzed data, and wrote the manuscript.

### Conflict of Interest Statement

The authors declare that the research was conducted in the absence of any commercial or financial relationships that could be construed as a potential conflict of interest.

## Supplementary Material

Figure S1 Number of fluorescent eGFP.vpr HIV-1 BAL virions in the FCS focal volume (~1 fL) as a function of input p24 concentration. The measurements were performed in triplicates and average values are shown. Error bars indicate standard deviations.
