# Supplementary figures and images for: Concurrent Exposure of Neutralizing and Non-neutralizing Epitopes on a Single HIV-1 Envelope Structure
Source: Front Immunol. 2019 Jul 5;10:1512. doi: 10.3389/fimmu.2019.01512 (PMC6628914; doi:10.3389/fimmu.2019.01512)

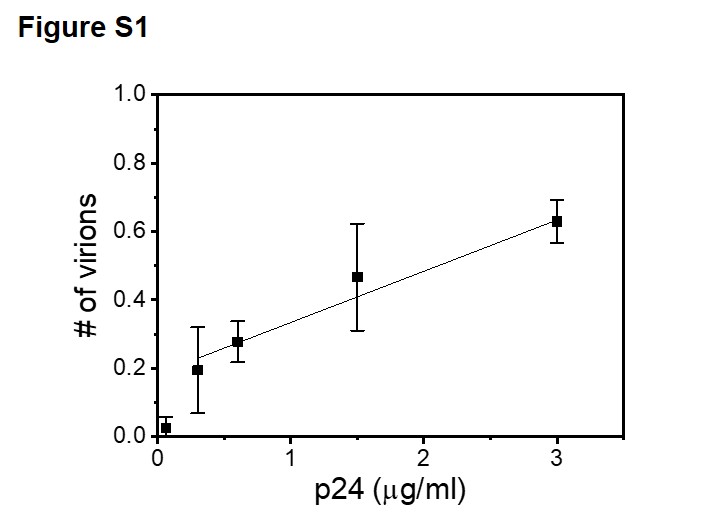

Supplement: Figure S1 — Number of fluorescent eGFP.vpr HIV-1 BAL virions in the FCS focal volume (~1 fL) as a function of input p24 concentration. The measurements were performed in triplicates and average values are shown. Error bars indicate standard deviations. [file Image_1.JPEG]
